# Psychoactive Comfort Products or Snacks: How Chinese Young Adults Perceive the Potentially Addictive Nature of E-Cigarettes

**DOI:** 10.3390/healthcare11101440

**Published:** 2023-05-15

**Authors:** Apei Song, Zihan Zhang, Zixi Liu

**Affiliations:** 1 School of Law, Society, and Criminology, Faculty of Law and Justice, University of New South Wales (UNSW), Sydney, NSW 2052, Australia; apei.song@unsw.edu.au; 2 School of Sociology and Anthropology, Xiamen University, Xiamen 610225, China

**Keywords:** e-cigarettes, understand, young adults, addiction, snacks, public health

## Abstract

The potential health value and pitfalls of e-cigarettes are currently under dispute in the scientific community. Exploring young adult e-cigarette users’ perceptions would assist in adding a public dimension of understanding to the literature and in scientific public health decision making. Therefore, in this study, we collected and analyzed data from interviews with young adult (*n* = 14) e-cigarette users and found that many referred to e-cigarettes as “snacks,” indicating that they considered that both their frequency of use and addiction were manageable and that they could stop using e-cigarettes at any time. To further understand the behavior of Chinese young adults in relation to their perception of e-cigarettes as a “snack”, the study developed a social context framework (crossroads model) and psychological judgment model to explain how youth e-cigarette users’ perception of “controlled addiction and ready cessation” arises. These models can be used to assess the effectiveness of e-cigarette policy.

## 1. Introduction

In recent years, e-cigarettes have gradually become popular in various countries. These devices, which produce aerosols by heating a liquid containing a variety of chemicals such as nicotine, additives, and flavorings, have become increasingly popular among young people, with the number of e-cigarette users in this demographic having grown rapidly in recent times.

The Sixth Conference of the Parties to the World Health Organization Framework Convention on Tobacco Control report detailed that the global expenditure on e-cigarettes was USD 3 billion in 2013. Without considering trends towards banning them, e-cigarette sales are expected to increase by 17 times by 2030 [1], with the e-cigarette industry predicted to become one of the eight major global industries [2]. China’s e-cigarette industry is also growing rapidly. By 2020, the national penetration rate of e-cigarette users reached 1% [3], with sales of more than USD 20 billion recorded, as the marketing of small smoke products with bullet replacements became mainstream. According to data from the Chinese Center for Disease Control and Prevention, the e-cigarette use groups in 2018 were dominated by young people, with 1.5% of e-cigarette users being people aged 15–24 years old, and the proportion of those who had heard of or used e-cigarettes and were now using them having increased compared with 2015. In 2019, youth e-cigarette users in China increased to 2.7%, with 3.55 million people having been added that year [4].

Early e-cigarette products were marketed as smoking cessation products and were supported by some tobacco control organizations because they followed the global political trend of tobacco control [5]. Thus, the “alternative” value of e-cigarettes has been the focus of scientific studies. Previous studies examining people’s level of dependence on cigarettes and e-cigarettes have reported a tendency for the two types of dependence to alternate, i.e., to ebb and flow [6], thus confirming the alternative value of e-cigarettes with regards to cigarette cessation [7,8,9]. Such studies are also taking place in China; these have examined the health risks, development trends, market supply and demand, and regulatory measures of e-cigarettes based on their alternative value [1,10,11,12,13,14]. Recently, some scholars have used Machine Learning and Artificial Intelligence to further examine usage behavior [15,16]. In addition, recent emphasis on the addictive, social, and flavor appeal aspects of e-cigarettes has framed the devices as a new tool for fashionable socialization [2,17,18,19,20]. This means that, for e-cigarettes, portrayed marketing terms and familiar usage environments also lead to the generation and transmission of usage behavior.

In the context of the parallel emphasis on e-cigarettes’ alternative role in smoking cessation and the scientific knowledge regarding their taste risks and potential health dangers, how exactly do many youth consumers perceive e-cigarettes as a substance? Is the public ignorant of the risks of e-cigarettes, blindly following the hype surrounding these devices, and if so, will this ignorance evolve for many users into the addictive dependence that is so worrisome to academics? This study took young adult e-cigarette users as its research group and focused on “how users understand ‘e-cigarettes’ and their behavior.” Firstly, we assessed young adults’ understanding of e-cigarettes, and then explored how this publicized cognition is formed from the comprehensive perspective of the social environment and psychological judgment. Next, we returned from public knowledge to scientific knowledge, theorizing and logically establishing a model and framework for understanding the research group’s behavior and empirical cognition in order to facilitate public health policy and support organizations that assess individual e-cigarette use behavior.

## 2. Literature Review: Scientific Discussion for E-Cigarettes

### 2.1. An Alternative or Not?

One of the main differences between e-cigarettes and tobacco lies in their safety of use. How are e-cigarettes a safer alternative to smoking? Their safety is demonstrated firstly in their inhibition and reduction of combustion, which studies have proven to be one of the leading causes of tobacco-related morbidity and mortality [21,22], and secondly in their reduction of the environmental nuisance caused by second- or third-hand smoke. As e-cigarettes produce less toxic vapor than smoke from combustible cigarettes [21], some scholars consider them as being relatively less harmful to health than tobacco [23].

Another difference is that e-cigarettes function as an alternative to tobacco during smoking cessation. The relatively low risk of e-cigarettes makes them an essential aid in quitting smoking. They are the most popular method of quitting in the United Kingdom and the United States [24]. The health concerns of tobacco (nicotine) addiction are of great concern [25] and can lead to physiological and psychological problems. Physiologically, nicotine increases dopamine, produces adrenaline and blood glucose, and for a short time, brings pleasure, increases concentration, reduces appetite, and increases energy, but this good state does not last; a bad mood returns, followed by a greater craving for pleasure, which leads to the vicious cycle of tobacco addiction [26]. When an addictive substance is used repeatedly, tolerance to the substance gradually develops, which leads to demand for higher quantities of the substance in order to achieve the same effect [27]. Regarding the multidimensional and complex self-control of tobacco addiction, prevention, treatment, and cessation are of great concern [28,29]. Compared to other ways of quitting, e-cigarettes offer a pleasant alternative to smoking that can meet the needs of smokers by replacing the physical, psychological, social, cultural, and identity-related aspects of addiction [30,31].

However, the alternative effects of e-cigarettes are still worthy of deeper investigation. Studies have found secondary effects, such as smoking relapse, dual use, and priming effects in e-cigarette users [31,32]. Meanwhile, opponents argue that e-cigarettes and traditional cigarettes do not have a causal relationship, and that the temporary, linear channel between them is interfered with by other factors. Further, they argue that e-cigarette and traditional cigarette use behaviors are understood as sequential connections, and that these behaviors are part of a complex holistic process that also includes risk behaviors, consumption, and a variety of social complexities (gender, race, class) that cannot be reduced to individual components [33].

As a new tobacco and recreational product, e-cigarettes have revolutionized traditional smoking behavior with their unlit, portable, and flavorful features. The use of e-cigarettes is rich in connotations, both as a form of smoking cessation and as a new form of smoking behavior, which stems from the cross-cutting attribute of e-cigarettes’ being understood as both “cigarettes” and different from “traditional tobacco.” As the number of e-cigarette users increases globally, the social implications of e-cigarette use must be taken into account. From an e-cigarette user’s perspective, how are e-cigarettes viewed, and do they have alternative uses, dual uses, and introductory effects? How do e-cigarette users understand e-cigarette behavior in their daily lives? This study will reveal how e-cigarettes are defined, used, and narrated in real social life.

### 2.2. Risk for Adolescents: Flavoring

As individuals enter adolescence, along with experiencing the awakening and strengthening of self-awareness, they can become susceptible to the influence of peers, popular culture, and fashion discourse, and can develop health-hazardous behaviors, such as e-cigarette use [34]. A total of 3.6 million young Americans used e-cigarettes in 2018, and this figure rose to 5 million in 2019 [21]. In 2022, the Annual National Youth Tobacco Survey, which focused on middle and high school students, found that more than 2.5 million U.S. students currently used e-cigarettes. Meanwhile, recent data from the Chinese Center for Disease Control and Prevention reported that young people had an e-cigarette usage rate of 1.5% in 2018 in China. By 2021, young people’s usage rate had reached 2.5%, which was higher than that of 1.6% among adults, a finding which has also been supported in recent empirical studies [20,35].

Research on youth e-cigarette use behavior has first focused on the causal factors leading to e-cigarette use, specifically the peer effect [2,20], social attributes, and curiosity drive. The peer effect refers to e-cigarette use that is influenced by friends, classmates, and family members [21]; following and imitating are the main behavioral factors of e-cigarette use in the context of peer relationships. Closely related to peer influence, the social uses of e-cigarettes relate to their use in a broader sense, with e-cigarette features such as their providing freedom from smoke-free environments and transmission sharing [36,37,38] reinforcing connections among young users in specific settings. The curiosity-driven psychological factor relates to the value of e-cigarettes as a fashionable entertainment and lifestyle exhibition tool, providing a subcultural lifestyle that satisfies young people [2]. However, this kind of psychological motivation is vulnerable to the manipulation of e-cigarette suppliers, and supply advertising can enhance the e-cigarette acceptance of young people through the use of attractive photos, videos, and text on online platforms where youth gather [21,36].

Second, the influences of the technology, taste and decorative features of e-cigarettes can be explained from a developmental and objective perspective. E-cigarette technology has evolved through four generations to achieve more powerful features and to feature adaptive flavors. The current fourth generation of e-cigarettes, the pod-style e-cigarette (pod system), has gained new highs in the number of young users due to, among other factors, its portability due to its low weight, improved oil leakage, and lower manufacturing costs and prices (USD 43−46 in the Chinese market) [39]. The adaptability of e-cigarette flavors avoids the olfactory irritation and tarry taste of smoking traditional cigarettes and is dedicated to a pleasant sensory experience for users while suppressing the aversive effects of nicotine [40]. Worryingly, the presence of flavor, while masking the irritation of traditional cigarette use, can likewise reduce the body’s immediate perception of harm. For instance, Pepper [41] and other scholars found that American adolescents preferred to try menthol-, fruit-, and candy-flavored e-cigarettes, a preference that led to almost six times the interest in trying fruit flavored e-cigarettes compared to tobacco-flavored e-cigarettes, which fuelled e-cigarette use (this preference was discovered also in Jongenelis’ study [42]). Finally, the decorative features of e-cigarettes also appeal to young people. Featuring shapes full of design elements that support personalized design, such as protective cases and decorations, e-cigarette devices reinforce their users’ desire to purchase e-cigarette products [21,43,44].

Therefore, researchers have grown concerned that e-cigarettes can trigger more complex negative effects than traditional cigarettes. First, adolescents and young adults do not have a clear understanding of the hazards of e-cigarettes and only perceive them to be less harmful than traditional cigarettes [40], which could lead to physical dangers for users. Second, people who have tried e-cigarettes are more likely to use traditional cigarettes, given the “introductory effect” of e-cigarettes [45]. A meta-analysis of 91,051 adolescent subjects showed that the risk of adolescents using e-cigarettes and then using traditional cigarettes was 2.21 times higher than the risk of “never using e-cigarettes” [46]. Therefore, it is likely that e-cigarette addiction will occur before a user quits traditional cigarettes [47]. Based on this perception, some regions and countries have controlled the sale and use of e-cigarettes by improving state laws, regulating sales, and publishing the hazards of e-cigarette ingredients [48,49,50].

Therefore, our study will add to the complex debates surrounding and scientific understanding of users’ perceptions of e-cigarettes, particularly their understanding (or misunderstanding) of the addictive nature of the substances involved, in order to bring individual awareness and perceptions into scientific consideration and reflection. In this study, Chinese young adults were selected as the subgroup of the study group in order to step outside of the bilateral family and school relationships that underage groups are limited to [51] and to further explore how and why a dynamic, complex, typical daily behavioral activity changes in a more complex social context. Further, we focused on young adults (17–35 age group), who are at a stage of emerging independence and maturity and are experiencing expanding social relationships and fundamental economic independence, in order to explore the expressions, understanding, and meanings under the surface of e-cigarette user behavior.

## 3. Method

### 3.1. Preliminary Preparation: Survey

The study mainly used a mixed method that combined questionnaires and interviews. Initially, in December 2021 and from January to June 2022, we advertised the questionnaire primarily online, and eligible youth within the age restriction (17–35 years old) were invited to take the initiative to fill in the questionnaire. During this period, we conducted three recruitment campaigns (Supplementary Materials), in which 135 respondents were recruited, and 132 questionnaires were completed. The main purpose of the questionnaire was to understand the essential use of e-cigarettes among youth, with various information being collected, including demographic information, the respondents’ habits and frequency of e-cigarette use, their access to information, and their self-assessment of the relationships between e-cigarettes and traditional cigarettes, health, and attitude. The specific tests and data relationships are accounted for in Supplementary Materials.

### 3.2. Interview

We then recruited interview participants, and a total of 30 e-cigarette users accepted the interview invitation.

Starting in May 2022, we conducted interviews with the voluntary participants. The interviews were divided into initial interviews, in-depth interviews, and post-return supplements. The initial interviews were conducted from March to June and focused on e-cigarette use habits and storytelling experiences. The in-depth interviews were conducted from the beginning to the end of June. Based on the initial interviews, 14 respondents were selected for the in-depth interviews based on the richness of their e-cigarette use habits, the depth of their practices, and the variability of their experiences, with the in-depth interview times ranging from 20−40 min. In early July, after primary and secondary coding of existing interview materials (the coding process is accounted for in Supplementary Materials), we conducted brief additional interviews with some interview participants, which were eventually stopped due to saturation of interview information. The interviewee information is shown in Table 1.

## 4. E-Cigarette Use and Experience Narrative

Vaping has gained popularity among youth in recent years, yet this behavior is considered as smoking and therefore an “unhealthy pursuit” [52]. What about the use of e-cigarettes among young adults? What do e-cigarettes mean to them, and do they represent addictive products that can lead to substance use disorders (SUD)?

First, through questionnaire analysis, we assessed e-cigarette use among the 130 questionnaire respondents and made a macro-level overview of their usage (data analysis in Supplementary Materials). Second, the perception of e-cigarette use among young adult users was interpreted qualitatively based on the interview materials.

Thus, through the analysis of the 130 questionnaires, the relatively macro-use behaviors of young adults can be understood as follows: youth know about e-cigarettes and how to purchase them. Some of the respondents reported having personal experience with e-cigarette use, with there being a high number of users within the last 2–3 years, and the dose of use was reported to be mainly medium to high frequency (1–2 puffs in a month). The effects of gender, income, education, and age differences within the group were insignificant (limited by the sample size), and all respondents showed signs of use. Additionally, and more noteworthy regarding the correlation analysis, is why these youth e-cigarette users still exhibited use behaviors and expressed perceptions of e-cigarettes being manageable, healthy, and safe when they knew about their adverse effects.

Further, in several in-depth interviews with young users, in addition to describing e-cigarettes as addictive and stress-relieving psychoactive comfort products, the interviewees suggested that e-cigarettes hardly played a role of releasing stress and served more as a snack in their daily lives. The term “Snack” is used to describe e-cigarettes because snack food has specific temptation characteristics but is also relatively controlled and safe.

The interviewees described e-cigarettes as similar to spiritual comfort items, with this being mainly closely related to their initial individual e-cigarette use intentions. Among the respondents, the first use of some users was primarily to cope with stressful events in their daily life, including, but not limited to, love disappointments, exam stress (such as gaokao [53]), and conflicting family relationships:

*“October 2019, I remember this day, I failed to confess my love, and then my friend was vaping, so I borrowed it and vaped it, until now.”*

*(Xiaoyu 20220617)*

*“Because of the pressure before the examination, (I) smoked the traditional cigarette first, and then feel tabacoo uncomfortable, choose the e-cigarettes. Also, e-cigarettes is the fashion.”*

*(Aman 20220614)*

*“Parents quarrel, I am no way to cope, so always avoidance (and) hiding. I just found this (e-cigarettes) to try., I’ve been vaping, maybe want my parents to focus on me.”*

*(Wasi 20220618)*

The interviewees noted that they chose to use e-cigarettes because the low nicotine content in e-cigarettes can play a role in relieving stress and eliminating pain. In addition to their reasons for initial use, the interviewees generally discussed the status of their e-cigarette addiction, suggesting that their behavior meets some of the characteristics of substance addiction. For example, they described craving and having a strong desire to use e-cigarettes and using larger doses than they expected or using them for more extended periods. Further, they judged e-cigarettes to be a product that has some mental impact, that is, a psychoactive comfort product.

*“Addiction definitely, I’m addicted. I put it in my hand when I write, and I will use it a lot if I don’t pay attention and keep vaping. ”*

*(Xiaoyu 20220617)*

*“Vaping* *, I have not been denying this inhaled, having nicotine, it is harmful to the body.”*

*(Hetian 20220602)*

*“I also recently a week I inhaled very often, I did not figure out why. Recently, I had to know where it was, and then I had to keep it on hand, so I didn’t know when I wanted to take a sip.”*

*(Maimai 20220302)*

However, the respondents also repeatedly referred to e-cigarettes as “snacks” and described how they found the variety of flavors they offer appealing. Although they emphasized the addictive nature of their e-cigarette use, they stated that this was not a symptomatic behavior of substance addiction disorder but rather a snacking behavior. This snacking use was characterized by the following features: “Habitual craving for use but accompanied by intermittent interruptions,” “Ready adjustment of dose and duration of use,” and “Awareness of health risks and occasional quitting.”

“Habitual craving for use but accompanied by intermittent interruptions” refers to how the interview participants described stockpiling some e-cigarettes and developing a habit of e-cigarette use because of daily use. Specifically, they reported using e-cigarettes at a fixed time after writing, eating meals, or exercising (this behavior is similar to eating popcorn while watching a movie). After using e-cigarettes for a long time or over a high-frequency period, the interviewees reported developing a sense of disgust for the taste of e-cigarettes and their oral odor and consciously controlled their use.

*“Vaping has to relieve hunger well* *, a little bit like eating bubble gum.”*

*(Ajing 20220620)*

*“ When e-cigarettes are vaped too much, you will be a little dizzy and nauseous, too much nicotine intake. The e-cigarette is very easy to cause this situation because of no unpleasantness (tar feeling), you do not know how much to vape...”*

*(Hetian 20220602)*

*“The e-cigarette with the kind of flavor, after vaping more, will make you feel a little nauseous, chemical feeling is powerful.”*

*(Aman 20220614)*

The second feature was “Ready adjustment of dose and duration of use.” In addition to occasional dose control due to taste aversion, the participants reported adjusting their quantity of e-cigarette use due to their current social situations or goals, such as college entrance exams, intimate relationship maintenance, pregnancy preparation, weight loss, etc.

*“I was able to control it... vaping has a big effect on your body, and if you’re thinking about pregnancy, you definitely need to quit e-cigarettes, so I tried to quit and then I found it fairly easy.”*

*(Aman 20220614)*

*“My ex-boyfriend didn’t care if I smoked or not, and then I would use his e-cigarettes. Now my boyfriend doesn’t let me vape, and I’m getting older so start to quit.”*

*(Xiaoyu 20220617)*

“Awareness of health risks and occasional quitting” was also commonly mentioned in the interviews. The participants, especially those who reported more than two years of e-cigarette use, all indicated that they could perceive the health risks associated with e-cigarettes.

*“If I vape more, I feel a little uncomfortable, dizzy, a little sick to my stomach, always not very good for the body.”*

*(Wasi 20220618)*

*“ I felt it was firm to the stomach that irritation, plus my digestion was not very good during that time. After vaping feel bloated and very uncomfortable, so that period, I thought, do not vape.”*

*(Xiaoqi 20220531)*

Some participants reported having already quit; some even had stopped for three months and did not desire to intentionally use e-cigarettes again. Interestingly, their quitting e-cigarettes was described as similar to not eating snacks, as the participants related using e-cigarettes (snacks) when offered in a particular setting but that it did not trigger addictive use again.

*“Surely addiction but having tried to quit. Just deliberately not bringing it with you, or not allowing the device to be in front of you, naturally don’t think about using it, and it’s not particularly hard.”*

*(Aman 20220614)*

*“You sometimes go out with other people and see other people there vaping. But because I put that cigarette stick in school and didn’t get it back, I control myself.”*

*(Tongtong 20220607)*

The snacking attribute of e-cigarettes was more prominent in the participants who also reported using traditional cigarettes. The participants who used tobacco stated that e-cigarettes only make up for the inconvenience of being in no-smoking places. Further, they described how their low nicotine content and elimination of tar reduce the pleasure of smoking, and thus they will still choose traditional cigarettes when the occasion allows for their use. They therefore evaluated e-cigarettes as a “better than nothing” snack.

*“For the smokers who use traditional cigarettes themselves, e-cigarettes are considered to be a bit useful in terms of portability, smoking ban places can be comforted by electronic cigarettes, it (e-cigarettes) nicotine content is not enough, the taste is too sweet, there will be a sense of plastic, strictly speaking, I do not like, but special circumstances mouth greedy words is also better than nothing.”*

*(Alee 20220620)*

This narrative of youth perceptions reveals that e-cigarettes do not have a single material identity at the cognitive level. Their psychoactive comfort goods attribute relates to the medical definition of e-cigarettes and the addictive properties of the substances within them themselves. However, e-cigarettes’ snacking attribute shows that beyond materiality, and considering e-cigarettes’ mutuality and relationship shaping regarding the user, e-cigarettes are social products with realistic, expressive, diffuse and cultural symbols. As Shérazade and colleagues pointed out, the “Reasons for using e-cigarettes in young adults are varied and are not limited to stopping smoking.” [54] In the present study, as shown by the narrative’s emphasis on e-cigarettes’ snacking attribute, the young adult participants declared their control over their e-cigarette use and the products’ relative safety regarding substance addiction concerns, which in turn led to different reported behaviors, such as the participants’ frequency of use, dosage, relapse, and withdrawal.

## 5. Understanding Young Adult Users’ Cognitions and Behaviors

In the previously described results, why were two perceptions reported, especially the perception of e-cigarettes’ snacking properties, which differ from the mainstream medical and scientific view? The cognitive differences in the reported results suggest that developing an understanding of inhaled nicotine/e-cigarettes cannot proceed solely from a substance perspective but should also occur within a complex social framework and with consideration of individual psychological dimensions. In this way, how this difference arises and what causes it to exist can be uncovered.

By developing both social and psychological dimensions, we next categorized the complexity of young adult perceptions and behaviors regarding e-cigarette use into exogenous and endogenous dynamics. The exogenous dynamics refer to the crossroads at which young adults find themselves in their social environment, while the endogenous dynamics analyze the decision processes that influence youth e-cigarette use through six-dimensional psychological criteria. This approach is related to Hughes’ multilinear connections perspective [33], which calls for studying multiple, multidirectional, and multilinear connections between various “elements” of the user, but also for avoiding a deep dive into a casual collocation analysis where infinite factors are connected, i.e., “it’s all connected”. Even though there may have been interference or confusion in the order of connections, we tried to summarize and organize the information obtained in the study in order to present a logic that would be more conducive to cognitive understanding. At the same time, we were alert to the fact that factors outside of this exogenous and endogenous logic are also associated with youth use behavior.

### 5.1. External Motivations: The Crossroads Situation

**Figure 1:**
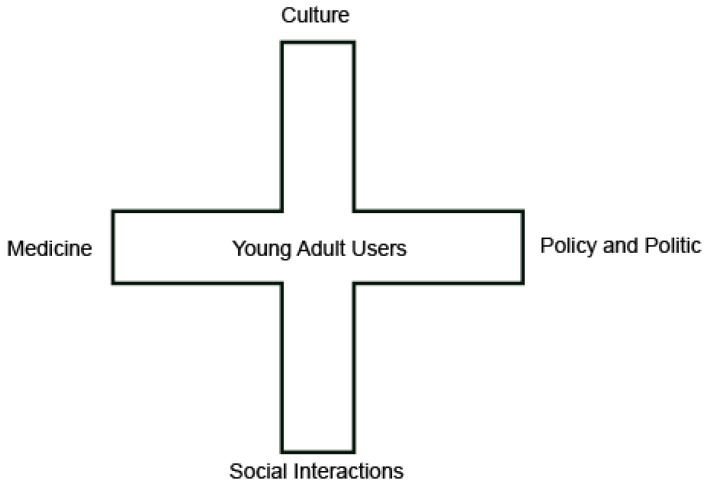
The Crossroads Situation for Young Adult Users.

In this study, placing nicotine/inhaled nicotine into a social framework in order to understand user behavior revealed that certain cognitive logics and use behaviors, such as harmfulness or harmlessness, dose and frequency of use, and abstinence, are born out of relatively complex contextual and relational considerations (findings that are similar to those of Hughes’ study [33]). Through analysis of the questionnaires and interviews conducted, we interpreted youth use behavior along four social dimensions: medical, social, cultural, and political. Through this, we mapped out a concept called the social situation at the crossroads, as shown in Figure 1. This crossroads situation is a relatively literary concept, but it vividly explains how youth at the crossroads are wrapped up and influenced by these four forces, and graphically illustrates how changes in any one of these forces affect the perceptions and behavior of young adults.

As shown in Figure 1, the four social forces involved in this crossroads are addiction discourse, which is represented by medicine; interpersonal relationships, represented by social interactions; e-cigarette subculture, represented by culture and its relationship to mainstream culture; policy and politics, defined by regulatory programs and laws targeting e-cigarettes.

#### 5.1.1. Medical Discourse

Medical discourse on e-cigarettes frames them as an alternative to tobacco (alternative methods and devices) [55]. In countries such as the United Kingdom, Australia, and the United States, e-cigarettes play an essential role in tobacco cessation [24,56]. Medical researchers are currently exploring the health hazards of e-cigarettes [57], including but not limited to their potential to trigger lung, bronchial, and cardiac problems. However, our survey of youth e-cigarette users in China found that the respondents were generally aware of the addictive characteristics and health hazards of e-cigarettes, even though they still used them. However, some respondents reported still mixing traditional cigarettes with e-cigarettes, while others never used traditional tobacco before using e-cigarettes directly, with the substitution effect of e-cigarettes regarding smoking cessation not being apparent in these cases.

As mentioned in Section 4, many participants reported using e-cigarettes as snacks, and while they acknowledged the unavoidable adverse effects of e-cigarettes and their addictive nature, they expressed that they could control their use.

*“Health concerns, obviously because of the knowledge that this thing has nicotine will have an impact on (health), so I control use times, circumvent some health risks.”*

*(Xiaoyu 20220614)*

*“I think no matter what it is, addictive is not good. this amount is now the upper limit of three cigarettes bomb a month, and if it exceeds this frequency, I will not buy.”*

*(Maimai 20220302)*

This idea of “Controlled addiction” among e-cigarette users differs from the medical assumption that e-cigarette addiction is out of control. The documented youth response to the substitution effect enriches the single idea chain of “tobaccos—e-cigarettes,” and the participants’ practical use of e-cigarettes included replacing traditional cigarettes with e-cigarettes, using a mix of tobacco and e-cigarettes, and directly using e-cigarettes.

*“I feel completely replaced. No e-cigarettes, okay, but no cigarettes, I do not feel good. I have been in Shenzhen for 21 days in isolation. For the first few days, I am vaping. I need to say it is not an alternative, only a supplement. That means, smoking is probably important for me than vaping.”*

*(Hetian 20220614)*

*“I think the replacement is a process, the original you mainly use cigarettes, and then use e-cigarettes, we can perhaps say in this case is a replacement, but there is a meaning, this is your own choice, not because all cigarettes are out of stock, I can only vape.”*

*(Xiaochen 20220619)*

*“My friend in the United States, who took me to smoke e-cigarettes, itself from the traditional cigarette to go over, is considered to replace the successful one.”*

*(Haluo, 20220608)*

Medical discourse, being at one end of the crossroads outlined above, influences e-cigarette users’ perceptions and behaviors through the power of knowledge, and in particular through the scientific exploration of e-cigarettes and their hazards. Inevitably, however, changes to these users’ perceptions and behaviors will be caused by influences outside of this discourse.

#### 5.1.2. Social Interaction Dimension

Social networks or interaction ports, and interpersonal relationships more generally, exert an influence on e-cigarette use [58]. In this study, the social effects experienced by the participants differed slightly from those associated with the interpretation of e-cigarettes as a social tool [59]. First, in a similar way to social tools, the participants reported occasionally reinforcing social connections with peers through e-cigarettes.

*“It depends on what kind of occasion, if t a group of people are more fun-loving, and then all vape, you also vape, there may be a topic of entry point, or sometimes we will taste each other. although it is dirty, I am not reasonable to reject because they are friends.”*

*(Aman, 20220614)*

*“Go to a place like a bar, talk about the packaging of the electronic cigarette, the feel of the stick, and so on, other times less will be the topic of e-cigarettes.”*

*(Xiaochen 20220619)*

However, on most occasions, the interviewees reported deliberately avoiding their use behavior to avoid causing secondary harm to others. Further, they noted how they did not want those they were in close relationships with, especially their parents, to know about their e-cigarette use behavior.

*“The boy in the dormitory building would use his whole hand to cover the e-cigarette and then vape it tightly to his mouth. It’s quite a covert way to vape, and he may not be willing to let others know that he is vaping, and so am I. You don’t have any benefit in announcing that you’re vaping; it’s unnecessary for me.”*

*(Haluo, 20220608)*

*“I am careful to smoke and avoid my relatives. When my mom caught me smoking e-cigarettes before, they would strongly urge me to quit, send me the news about the dangers of e-cigarettes every day, and then limit my living expenses.”*

*(Xiaoyu, 20220614)*

Social interactions influence use behaviors in different contexts and settings by facilitating socialization, reinforcing relationships, and managing impressions. E-cigarette users may avoid family members and strangers who dislike the smell of vaping, and they may use them primarily in environments (personal or public spaces) where e-cigarettes are allowed and encouraged.

#### 5.1.3. Cultural Dimension

The cultural dimension of e-cigarette use has three main aspects: the recognition of and participation in the e-cigarette subculture [60,61]; the understanding of tobacco in mainstream culture and its acculturation [62]; the influence of gender [63,64].The development of e-cigarettes in China has been characterized by various changes to the products’ positioning and design, moving from their first iteration (Supplementary Materials Figure S1), where they were simulated cigarettes framed as smoking cessation alternatives, to the older kind of e-cigarettes, which gradually modified their form and began focusing on their taste (Supplementary Materials Figure S2), and finally to the independent e-cigarette products, which are framed as convenient and rich in taste (Supplementary Materials Figure S3). The development of e-cigarettes has attracted some young people to set up loose groups or communities to serve as purchasing channels, gradually forming “players’ groups” with specific subcultural characteristics; within these groups, some members are known as “hardcore players”. Tokle and Pedersen labeled this subcultural group ‘cloud chasers’ [61]. These behaviors give e-cigarettes a special meaning, and in communication, this meaning is recognized and spread, thus forming a cultural paradigm with certain sub-cultural characteristics. In this study, some participants acknowledged and participated in this kind of communication.

*“My earliest use e-cigarettes, from a store called Spartacus, they will organize the community, we communicate the type of machine, they call the e-cigarette “machine”, like people playing motorcycle, there will be a variety of aesthetic communication, what emphasizes the mechanical sense, comfort. Often use these ergonomic, mechanical words as if in the description of the upper motorcycle. They will also communicate the play, and the play refers not only to how to blend the taste but also a variety of evaluations, what the richness of the smoke, how much smoke out of the taste, is to measure a variety of physical indicators, these physical indicators are certainly with this meaning in, they determine a unique for this thing, each machine, give it a very anthropomorphic title, this is What is it? The Terminator, that’s Napoleon, that sort of thing.”*

*(Hetian 20220602)*

Not all participants recognized the existence of this subculture, and many asserted that e-cigarettes have a low cultural identity or that they had not considered the impact of e-cigarettes in terms of their cultural values.

*“It does not have the so-called cultural connotation in it, unlike sneakers cultural connotation, such as the AJ series worn by Jordan, there are some special colorways, in the special field, such as how many points he scored this game, what colorway, there is a cultural background in doing endorsement, and then you go to buy those replicas of this colorway to collect and even have a lot of glory in it. The e-cigarette is a single product that can be used as a snack and as an accessory (if you hang around the neck), but it is not supported by the people and stories behind the circle.”*

*(Haluo, 20220608)*

The participants who did not recognize the sub-cultural characteristics of e-cigarettes spoke of the cultural context of items such as sneakers, discussing their production, purchase, and communication attributes, while noting that e-cigarettes did not have a sufficiently attractive set of cultural associations. Instead, this segment of users reported viewing e-cigarettes only as daily necessities or snacks.

In addition, the understanding of tobacco in mainstream culture and the cultural connotations derived from this also influence e-cigarette use. Traditional Chinese cigarettes have a more mature cultural meaning than e-cigarettes, regarding providing access to the adult world and serving as a critical prop in “relationship” and “gift giving” activities.

*“Smoking is middle-aged feeling, while the e-cigarette’s more image younger.”*

*(Xiaochen, 20220619)*

*“Generally, people like our parent’s generation, begging others to do something, on the delivery of tobaccos as gifts. Wine is also okay, but e-cigarettes are not good.”*

*(Aman 20220614)*

*“You will have the impression that when your mother is unhappy, your mother will smoke, and when your father is annoyed, your father will smoke, is a pattern. This pattern is as if you learned it at a very young age, you know when you one day become like them, you may seek such a kind of, maybe this thing is always there, is something that can provide you with comfort.”*

*(Xiaoqi 20220531)*

Accepting some degree of pain is considered a sign of youth maturity and growth, and this is reflected in the ability to tolerate and endure choking during tobacco use. The tarry feeling and burning of the throat associated with traditional cigarettes both align with this pain-tolerant maturity. Thus, e-cigarette and tobacco users can be divided into young adults and middle-aged adults, respectively. In addition, compared to e-cigarettes, tobacco is more able to facilitate Chinese society’s expectations regarding asking for a job or giving a gift.

In the present study, the participants who reported mixing tobacco and e-cigarettes noted that there was also a hierarchy of tobacco use, mainly in terms of contempt and low acceptance. That is, this hierarchy is related to the predominantly adult social sample, the dominant culture, and the far-reaching nature of different types of nicotine use. Some participants said that they perceived traditional cigarettes to be superior to e-cigarettes, and that this superiority was not tangible but rather somewhat scattered throughout daily life.

*“They will be more superior to the trendy thing of the moment (e-cigarettes), it is something for kids to play with.”*

*(Hetian, 20220602)*

*“It feels like there is a gap where, if they smoke traditional cigarettes at the dinner party, I will follow, not quite yell at everyone to smoke e-cigarettes.”*

*(Xiaochen, 20200619)*

*“I think cigarettes are also really a definite grade thing. Sometimes e-cigarettes are at the lower end of the hierarchy, and when you are with young people, people may think how cool you are, but now the electronic cigarette seems to be children’s play in educated or mainstream mature people.”*

*(Xiaoqi 20220602)*

Gender culture limits whether women can freely and comfortably use e-cigarettes [65]. The young men surveyed in this study generally expressed the opinion that female use behavior did bother them, while the female users surveyed stated that they perceived e-cigarettes as being more female-friendly than cigarettes but that they would still hide their use behavior or quit entirely because of intimate relationships or stereotypes.

*“I label myself as someone who is also a gender equalist in society. I may think that what boys can do, girls can do. But when I see girls vaping, I feel a little bit uncomfortable, so maybe subconsciously, I still think vaping is a male privilege, but when I see the abstract of girls vaping on the movie screen, I feel so cool.”*

*(Hetian, 20220602)*

*“Seeing a girl vaping, I would still be more shocked, but I wouldn’t use it to judge her character. I would be shocked at the act of vaping.”*

*(Haluo, 20220608)*

*“Because I find that there are quite a lot of girls vaping inside young people, so I think it’s quite normal, we all have hobbies.”*

*(Xin, 20220621)*

In general, a youth’s use of e-cigarettes will differ according to their interest in the e-cigarette subculture, their recognition and understanding of the place of e-cigarettes in mainstream tobacco culture, and the influence of gender culture. Where a user recognizes the e-cigarette subculture, their frequency of use is likely to increase, and their user behavior will likely become more profound and systematic. Recognizing the superiority of tobacco in mainstream culture may encourage e-cigarette users to gradually attempt to enter adult culture by using tobacco. Further, the female stereotypes surrounding e-cigarette use will likely encourage women to participate in affirmative and firm use of e-cigarettes or otherwise decide to follow the rules and regulations regarding e-cigarettes and give up using these products.

#### 5.1.4. Policy and Political Dimensions

The policy and political dimensions of e-cigarette use relate to the regulatory programs and laws surrounding e-cigarettes. During the research process of this study, China’s Tobacco Management Monopoly Bureau (TMB) and the General Administration of Market Regulation (GAMR) published e-cigarette management measures that restrict the sale of e-cigarette flavors other than tobacco flavors. Specifically, on 11 March 2022, the State Tobacco Monopoly Administration published the Measures for the Administration of Electronic Cigarettes, which explicitly prohibits the sale of e-cigarette flavors other than tobacco flavors, a regulation that has been in effect since May 1. On April 8, the State Administration of Market Regulation issued GB 41700-2022, the mandatory national standard for electronic cigarettes, which imposes strict restrictions on e-cigarette flavors and set a five-month transition period, from October 1 onwards.

In this study, we asked the interview participants to discuss this new policy’s impact on their e-cigarette use behavior. Some interviewees reported that the external force of flavor control provided an opportunity for them to quit, while others said that they had chosen to stockpile some e-cigarette products and maintain good purchasing channels.

*“I will sometimes feel dizzy and uncomfortable after smoking. It indirectly reduces my frequency of use or the degree of my liking. I do not have any resistance, just let it go.”*

*(Xiaoqi 20220531)*

*“Because of the control, I added the merchant store in WeChat, a box before ¥60, now up to more than ¥70. I also bought ten boxes of hoarded. In May, the Policy was introduced, I will quit no longer use.”*

*(Haluo 20220608)*

*“Tobacco taxes earn too much, cannot reduce on tobacco, only on the e-cigarette sales control.”*

*(Hetian 20220602)*

By influencing the market, this new policy has led to changes in youth usage behavior. In the April-May period following the policy’s introduction, the participants reported two behavioral outcomes: continued use and discontinued use, depending on their circumstances. Then, in June 2022, when the market became slightly less regulated and e-cigarette flavors other than tobacco began to be openly sold again, the user behavior of some participants shifted again.

*“I helped a friend stock up on ten boxes of Dragon Well in April. But now you can rebuy it. There is no intention to go to hoard.”*

*(Xiaoyu 20220614)*

*“Although I can buy it, do not affect me. I have gradually stopped using it since April, and the process is not very difficult, now it is good to use a smoke bomb a month, the key is not to take the initiative to vape, and there is no idea to smoke again”*

*(Wasi 20220618)*

As our analysis of each of the four forces shows, changes to any of these forces, i.e., their expansion or reduction, will affect youth e-cigarette use behavior. However, in addition to the external social forces that change youth e-cigarette users’ perceptions and behaviors, these users’ judgment, as a type of internal motivation, is also worthy of attention.

### 5.2. Internal Motivation: A Hexagonal-like Basis for Judgment

As outlined above, youth at the crossroads will behave differently according to the varying influence of the four forces. However, these forces ultimately impacting an individual’s final actions inevitably involves them accepting these external forces and forming judgments that guide their behavior. Thus, exploring the psychological judgment factors that drive e-cigarette use behavior is the focus of this section.

**Figure 2:**
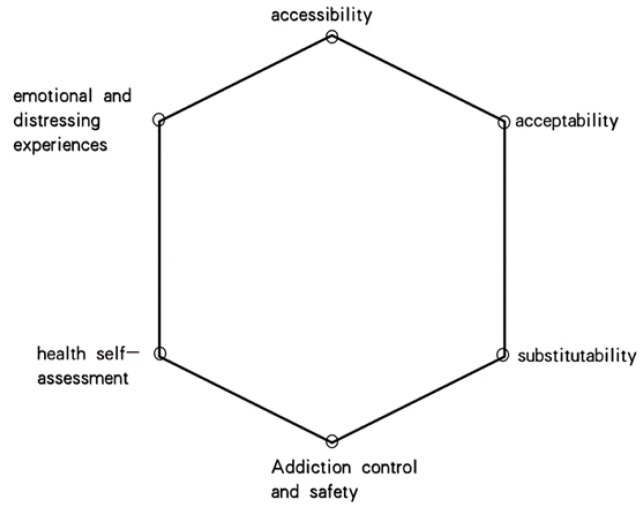
Hexagonal intrinsic judgment basis.

Through further regression analysis and assessment of qualitative data from previous studies, we categorized the psychological judgments that bridge external influences and behavioral outcomes into a hexagonal judgment model (Figure 2). This model involves six conditions that youth users consider, namely accessibility, acceptability, substitutability, addiction control and safety, health self-assessment, and emotional and distressing experiences. Although these six dimensions mean the model is characterized by a hexagonal shape, the relationships between these may be impacted when one or more dimension is met, such as the judgment of substance dependence symptoms [66].

Regarding the six conditions, as presented in Figure 2, accessibility refers to the ease of obtaining e-cigarettes, and is related to the cost of using these products. Accessibility factors, including the purchase of e-cigarette machines, cartridge flavor selection, purchase price, and portability, are generally subject to alteration by external influences, such as social policies, market changes, and user circumstances. As found in this study, if accessibility is made more difficult, some young users will exhibit the behavioral outcome of stopping their purchase of e-cigarette products and thus reduce or cease entirely their e-cigarette use after weighing the various conditions and price constraints (e.g., Haluo and Alee).

*“It this price is quite high, you if the normal channel is ¥99, I brought it online firstly, it was ¥61 a box, and then I added the WeChat to buy, the price is ¥70, not recently, and the canceled fruit flavor rose again to ¥80, no matter what, it is still costly. I am a student, the cost per month is too high. I will now gradually stop using it.”*

*(Haluo 20220619)*

*“I like the watermelon flavor. After the policy change, I had no stock on hand, and nearby downstairs stores were not available for sale. Friends later recommended me online shopping, but all needed to express 2−3 days. Now also the e-cigarette is used less and less.”*

*(Alee 20220620)*

Acceptability involves the acceptance of e-cigarette use behavior and information exposure and disclosure (this concept is similar to that of ambivalence in Majmundar’s study, and social acceptance concerns in Aubuchon’s study [67,68]). Our interviews found that some participants were able to avoid using e-cigarettes around family members and intimate partners, and some reported disliking using the products around strangers; they therefore described avoiding using e-cigarettes in public places where people are present. Acceptability generally involves medical and cultural influences that can produce behavioral outcomes such as avoidance and reduced use.

*“I vaped, and if they (parents) saw me, they would not give me money. I am not working now. I still need them to give me living expenses; they supervise me. First, they quarrel with me, then they educate me, and finally, they don’t give me money and don’t take care of me this way.”*

*(Wasi 20220618)*

*“Some people hang their e-cigarettes around their neck like a fashion item. They think it’s a little bit fashionable, but I think you don’t have any benefit to announcing that you’re smoking an e-cigarette. There’s no image plus, so that’s not necessary for me.”*

*(Xiaochen 20220620)*

*“In public, some people, even a ride, parking a moment to go out to vape, I will not. There is no need. I also do not have such a big addiction generally. In public places will not vape.”*

*(Maimai 20220302)*

Substitutability does not refer to replacing traditional cigarettes with e-cigarettes but rather to whether e-cigarettes play a role in replacing a product used in daily life. Suppose e-cigarettes can act as a substitute in certain situations, for example, during attempts to lose weight. In this case, and as found in this study, they can attract high-frequency use by women due to their sweet taste but lack of fat and calorie content (a finding that is identical to that of Morean [6]). As a result, the frequency and dosage of e-cigarette use during this period is enhanced accordingly.

*“Taro flavor is very popular with girls. I recommend this flavor to everyone. Smoke the first puff, all feel like eating dessert, I lost weight, a few days without dessert intake, its taste replaces the taste experience of dessert, very amazing. At that time, smoking is quite a lot.”*

*(Xiaoyu 20220614)*

Addiction controllability and safety refers to users being aware of their e-cigarette addiction but believing that they can control their dosage and withdrawal process, meaning that they always use e-cigarettes in a safe and controlled manner. This shows that e-cigarettes do not fit the traditional risk profile suggested by macro data [69]. This in turn indicates that substance dependence is always controlled by rational judgment. Further, addiction controllability and safety involve the differential expression of medical discourse and knowledge. In this study, some youth users acknowledged that they had a substance use disorder. They reported that they were convinced they could reduce their dose, quit, or relapse any time they needed to, with some participants already actively in the withdrawal stage or having quit successfully while experiencing no other intervening factors in the process. The only discomfort that these participants mentioned several times was there needing to adjust to the behavioral habit of vaping.

*“There was an addiction, but I tried to quit, and I was quite successful. I was able to control it, I was more repulsive myself, and smoking still has a greater impact on my body.”*

*(Aman 20220614)*

*“I quit e-cigarettes for three months, is just starting to take a smoke every day, and suddenly did not carry, the habit has a subconscious fall, you may want to ingest the nicotine at the beginning, and then it is not much feeling. The throat feels comfortable.”*

*(Alee 20220620)*

Health self-assessment, unlike controllability and safety, which relates to a user’s overall relationship with e-cigarettes, mainly refers to a user’s physical discomfort and self-perception of their health status. Health self-assessment involves the expression of medical discourse and knowledge under the logic of consent. Participants in this study reported reducing or ceasing their e-cigarette use for a period of time because of poor health, such as Hetian, due to breathing issues, Xiaoyu, due to the risk of pregnancy preparation, and Alee, due to a throat cough. Further, more negative self-assessments, such as Aman’s assessment regarding their stomach discomfort, led to active choices to quit (as seen also in Chen’s study [35]).

*“Recently, I often feel that my lungs are not strong, very uncomfortable, gasping for air, and then my body is a little weak. E-cigarettes very easy to cause this situation. I will use less in all these cases.”*

*(Hetian, 20220602)*

*“Coughing is also, probably after smoking for a month or two, you get that feeling. You can’t spit it out when you cough and feel like coughing up phlegm. after I stopped for a while, I found that it was much better, and then I thought, this is not very necessary, and then I am not vaping.”*

*(Tongtong 20220607)*

Emotional and distressing events refer to a user’s current emotions (positive or negative) and distressing experiences [70,71]. External social life events can bring stress [72], loneliness, sadness, happiness, and excitement, etc. These moods, and harrowing experiences in particular, can prompt users to use e-cigarettes heavily. Conversely, it is worth noting that some individuals use e-cigarettes more when they are in a happy or excited mood than when they are sad. In this study, Xiaoyu reported that they generally increased their e-cigarette use in response to the pressure of intimate relationship communication. Aman and Tongtong reported using e-cigarettes more frequently only when they had gained certain achievements. Xiaochen reported relying on e-cigarettes when memories of childhood stories emerge, and likewise, Xiaoqi reported relying on e-cigarettes to achieve mental stability and emotional detachment.

*“I recently am the mental state is not very good, is how to speak, in the medication, so sometimes the attack will feel, like a lifesaver, you grab it (e-cigarettes), and then it will make you think, I can still be stable here again. And then the specific description, I do not know how to describe, that feeling, that is, it will not let you calm, but not to collapse, is that I grabbed it.”*

*(Xiaoqi 20220531)*

*“I vape because feeling happy.You drink a little bit on the head. Vaping can make me sober a bit, feel more refreshing.”*

*(Tongtong 20220607)*

The six conditions discussed above are focused on exploring the successive changes that can occur in e-cigarette user behavior. While not only considering the influence of social forces on individuals (each condition is not one-to-one), they do explore how these forces guide e-cigarette users to make decisions, for instance, increasing or decreasing their dosage, quitting, and resuming smoking. Trade-offs are psychological processes that occur before judgment and decision making takes place. As these can vary according to individual differences, not all six conditions must be met, nor does there need to be a balanced mix of these conditions, for a user’s behavior to be influenced. As found in this study, youth e-cigarette users adjust their behavior when any of these six dimensions are met, and assign different levels of importance to these dimensions, with the dimension with the greatest weight potentially playing a decisive role in any behavioral changes (see Table 2).

As shown in Table 2, there were significant differences in the weights assigned to the six conditions by the different interviewees. If we set 100% as the total, each condition weighs an average of 16.7%. This variation allows users to make decisions and carry out actions with distributed differential trade-offs when social forces affect them.

The concept of the crossroads situation and the hexagonal judgment model discussed in this study provide pathways for interpreting youth cognition and behavior regarding e-cigarette use from both social and individual perspectives. As the two models are closely related, they can be considered in combination as providing a map for understanding differences in youth use behavior under temporal and spatial variations.

## 6. Discussion and Conclusions

*“The District Health Board said it had been a great tool to help quit smoking, and they were 95% less harmful than cigarettes.”*

*(a New Zealand news report)*

In June 2022, the Whanganui District Health Board of New Zealand announced that e-cigarettes were helping to make mental health wards smoke-free, and they described e-cigarettes as a good tool for smoking cessation, considering them as being 95% less harmful than cigarettes. Do e-cigarette users feel the same way? We explored this question in this study by conducting interviews and questionnaires. After compiling a narrative of young adults’ experiences, we found that e-cigarettes were perceived by youth users as a spiritual comfort item or snack. Further, the psychoactive attribute of e-cigarettes was found to be in line with the medical definition of the products themselves and their addictive nature. The snack attribute of e-cigarettes frames them as having practical, expressive, and diffuse symbolic cultural connotations that exist beyond the materiality of e-cigarettes as a social product, as these connotations interact with and are mutually shaped by users. At the same time, the narrative’s emphasis on this snack attribute implies that young users are aware of the controllability and relative safety of e-cigarettes, and also of concerns around their addictive nature. This perception was found to generate different behaviors in the participants’ daily lives, such as frequency and dosage, relapse, and withdrawal behaviors.

These two relatively static perspectives, while covering the diverse real-world cognitions and behaviors of youth users, involve both scientific perception and life experience. To further elucidate youth e-cigarette users’ process of cognitive generation, the study categorized their perceptions and behaviors regarding e-cigarette use as exogenous and endogenous dynamics. The exogenous dynamics involved a “crossroads situation” for the youth users in their social environment and were influenced by four social dimensions: medical, social, cultural, and political. The internal dynamics were analyzed using six psychological judgment conditions in order to examine the decision-making and trade-off processes of youth user behavior. The crossroads situation and the hexagonal judgment criteria provide pathways for interpreting youth cognition and behavior regarding e-cigarette use from both social and individual perspectives. The two models are closely related and provide clues for understanding the differential mapping of young adult use behavior under temporal and spatial changes.

The main contributions of this study, regarding both current theoretical and empirical research developments, include the following three points:

Firstly, the study enriches our perception of e-cigarettes at the public awareness level and offers insights from the Chinese context. For instance, in this context, it found that e-cigarettes are perceived by some youth users as snacks, and that e-cigarette use behavior is considered recreational. However, the hermeneutic view of recreational use is relatively macro and holistic. Both recreational and medical drug use is discussed in drug research, which enriches discussions surrounding drug practice and decriminalization from a drug user’s perspective. However, in this study, e-cigarette users were not found to exhibit non-recreational use behaviors. Therefore, instead of generalizing the view of e-cigarettes as being used recreationally, we should further explore the perceptions of and definitions used by e-cigarette users. Further, the emergence of the snack attribute of e-cigarettes dynamically and vividly describes the behavior of e-cigarette use in daily life as a “habitual desire to use but accompanied by intermittent aversion, readily adjusting the dose and time of use, and occasionally quitting when aware of health risks.”

Secondly, two correlated models are provided that assess youth perceptions and behaviors surrounding e-cigarette use. Considering the models in combination is sufficient to explain “why some youth use e-cigarettes and believe addiction is manageable despite knowledge of the harms.”

These models consider both social and individual perspectives and assess both external and internal motivations. Further, they assist in capturing empirical knowledge using analytical techniques, and also assist social workers in frontline addiction to understand and grasp the causes of behavior changes in e-cigarette users.

Through exploring external motivations, this study analyzed the contextual pathways of social forces and their combined consequences. Thus, we described adolescents as being at a “crossroads” of multiple connections, with this linkage formulation being similar to Hughes’ multilinear connections perspective [33]. We acknowledge the existence of multiple, multidirectional, and multilinear connections between the various “elements” of the e-cigarette user worthy of study and that practice forms part of an entire complex process. However, the multiple connections revealed by Hughes break through the double-chain single-line “channel” thinking of the Gateway, thus helping us avoid falling into inconvenient and vain metaphysical logic. Researchers using this concept need to organically combine and logically present all possible elements in order to provide a convenient tool for exploring causes and analyzing effects. The analytical processing that occurs on top of these connections leads to the emergence of combinatorial models.

Having a core focus on “how individuals make their logical trade-offs and decisions under the influence of social forces,” the internal motivation model explores six judgment conditions and considers the psychological process of young users’ guided behavior under different experiences and events. The model attempts to frame the decision-making and trade-off processes of e-cigarette users as forming a comprehensive and holistic set of conditions, therefore providing relatively complete and complex insights compared to some models that start with a single condition. In the user narratives, we also focused on the preferred meanings of different conditions for individuals, which helps to explain why seemingly illogical behaviors occur in situations where conditions present relative conflicts, such as confidence use behavior and controlled addiction prediction in youth experiencing harm.

Finally, unlike adolescent users, young adult users are free from the dual control of family and school and face a more complex social situation and more accessible standards of judgment. The concept of the crossroads situation is close to these users’ real life situation, in which they have no strong direct supervisors (e.g., parents and teachers in youth studies). These users need to grasp the consequences of e-cigarette use on their own, which are impacted by the integrated and complex dimensions of culture, policy, medicine, and socialization. In addition, instead of just experiencing the motive of temptation, young adults’ motives for e-cigarette use involve six critical perspectives, and these users possess the decision-making ability to choose to actively use or not use e-cigarettes. The study of young adults therefore provides rich insights on the experiences and differential thinking of this demographic.

This study had two limitations. First, screening young adults by age was the main criterion used, which perhaps excluded other potential participants who self-identified as young adults but were physically younger or older. Second, as the interviews for this study were conducted from May to June 2022, and the E-cigarette Regulation Notice in China was introduced at the end of 2022, the study did not assess whether e-cigarette users perceived changes in their e-cigarette use behavior after this regulation was declared. This will be considered in future research.

## Figures and Tables

**Table 1:** Interviewee information.

Pseudonym	Age	Gender	Years of Vaping	Dual-Use (Tobacco and e-Cigarettes)
Haluo	26	male	2−3	Yes
Wasi	25	female	1	
Xiaoqi	23	female	3	
Fan	23	female	1−2	
Hetian	24	male	5	
Maimai	23	female	1	
Tongtong	22	female	2	
Ajing	25	male	2	
Alee	19	female	2	No
Xiaochen	20	female	3	
Xiaoyu	23	female	2	
Aman	22	female	2	
Adong	21	male	1	
Xin	23	female	3	

**Table 2:** The relationship between hexagonal judgment conditions and the differences in their assigned weights.

Social Powers	Six Conditions	Interviewees and Assigning Weights (Average = 16.7%)
Policy/Politics Culture Society Medicine	Accessibility	Haluo (26%); Fan (50%); Ajing (30%) Adong (30%); Xin (30%)
	Acceptability	Wasi (30%); Tongtong (50%)
	Substitutability	Xiaoyu (24%)
	Addiction control and safety	Aman (29%); Alee (32%)
	Health self-assessment	Hetian (27%)
	Emotional and distressing experiences	Xiaochen (30%); Maimai (50%); Xiaoqi (55%)

## Data Availability

Not applicable.

## Supplementary Materials

The following supporting information can be downloaded at: https://www.mdpi.com/article/10.3390/healthcare11101440/s1, Files S1–S3.
