# Supplementary material for: Psychoactive Comfort Products or Snacks: How Chinese Young Adults Perceive the Potentially Addictive Nature of E-Cigarettes
Source: Healthcare (Basel). 2023 May 15;11(10):1440. doi: 10.3390/healthcare11101440 (PMC10218233; doi:10.3390/healthcare11101440)
Supplement: Supplementary file 1 [file healthcare-11-01440-s001.zip › healthcare-2267707-supplementary (1).pdf]

Supplementary

File S1

relevant posters for the recruitment and survey

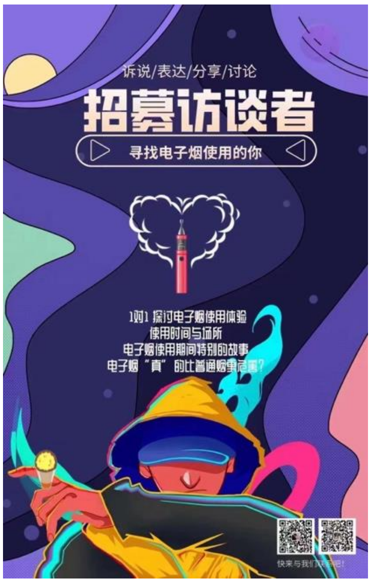

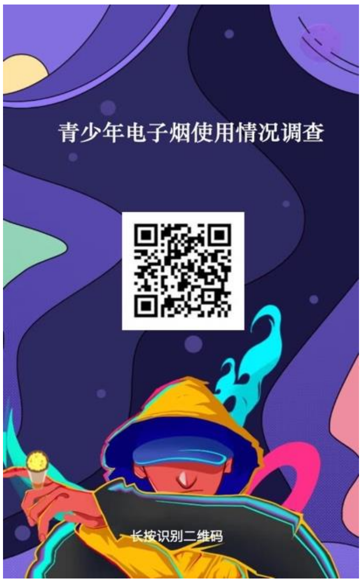

File S2

The reliability test of the questionnaire was (Cronbach. α) 0.893. The survey covered four parts:

- Basic demographic information
- Details of e-cigarette use
- Relationship between e-cigarette and traditional tobacco use
- Self-assessment

After statistical and correlation analysis, the primary data were compiled in Table S1.

Table S1: Survey on the use of electronic cigarettes

**Variables**	Statistical details (N_{total} = 130; $N_{e\text{-}cigarette\ use}$ = 60)	Correlation coefficient (with the variable of whether to use e-cigarettes)	Correlation coefficient (with e-cigarette attitude variable)	Correlation coefficient (with self-rated health after e-cigarette use)
**Basic information**				
Gender	male=76；female=54	0	-0.02	0.02
Age（limited 17-35）	Average=26	-	-0.28*	-0.18
Income	Average=5800 RMB	-	0	0.12*

(monthly)				
Years of Education	9yrs（0.77%）；12yrs（3.08%）；15yrs（3.85%）；16yrs（39.23%）; 19 yrs（50.77%）	0.22**	-0.33**	-0.20*
Location	urban=109；rural=21	0.29**	0.30**	-0.28**
Use e-cigarettes or not	yes=60；no=70		0.39**	-0.99**
Years of Using	Average=2.7 yrs；max=7 yrs Min= 0.5 yrs	-	-	-
Frequency of e-cigarette use in the past week	Very frequently = 14 (23.33%); Fairly frequent = 8 (13.33%); Fair = 16 (26.67%); Rarely used = 8 (13.33%); Not used = 14 (23.33%)		-0.31**	0.96**
**E-cigarette use details**				
Purchase sources	Offline brand counters = 43; online official mall = 12; online WeChat or facebook buying way = 24			
Using Brand	Relx=54；Yooz=1；Moti=8；Vvild=7；Boulder=3；others=9			
Flavor preference	Emphasis on specific flavors = 12; Preference for certain types of flavors = 42; No flavors preference = 6		-0.36**	0.98**
The use cycle of a bomb	One day = 8; three days = 19; one week = 15; one month = 10; one month and more = 5		-0.33**	0.96**
Frequency of Cigarette purchase	Three days to buy once = 3; one week to buy once = 8; one month to buy once = 27; one month and more to buy once = 22		-0.37**	0.98**
**E-cigarettes and tobacco use relationships**				
When did you use cigarettes	Before using e-cigarettes = 52; After using e-cigarettes = 3; Never use = 5		-0.38**	0.98**
Whether to dual use (e-cigarettes and tobaccos)	Yes=31; No=29		-0.39**	0.97**
**Comprehensive Self-Assessment**				
Self-assessment of health after using e-cigarettes	Healthier than before = 11; Less healthy than before = 15; No difference = 34		-0.34**	
Attitudes (Likert scale)	Very positive (5) = 4; more positive (4) = 9; neutral (3) = 68; more negative (2) = 24;			-0.34**

	very negative (1) = 25
C(Cronbach.α)=0.893； * p<0.05 ** p<0.01	

File S3

Data cleaning and organizing process

First, we transcribed and verified the collected data. In the process of transcribing the audio recordings, we also blurred the anonymity of the interviewer and the prominent third-party information and address information, and during the verification process, we verified the transcribed material word by word with the data from the audio recordings, marked the emotions of the interviewer in the audio recordings, and added some unspoken information through the context.

Secondly, after completing the verification of the interview materials, we conducted a coding process of "conceptualization - genus categorization - identification of core genus - establishment of theoretical analysis framework "and divided it into two coding stages. The first stage is the open coding stage, which mainly decomposes, examines, compares, conceptualizes, and categorizes the interview materials, including labeling, categories, attributes, and dimensions. The second stage is the main axis coding, which regroups the categories and identifies the core categories according to the framework of "situation-phenomenon-representation (representation practice)-condition-action." Based on the primary and secondary coding, we systematically link the core genera to other supporting genera based on the combined research questions and current theoretical contributions to present more exploratory findings. An example of the analysis process for the first step of coding is shown in Table S2:

Table S2: Coding process of interview (Heian) materials (fragment):

Original information after verification (excerpt)	coding			
	Labeling	Genus	Attribute	Dimension
Yeah, they often use ergonomic words and ome mechanical words. Then it's like a little bit of a motorcycle in the description, they also communicate the play, which refers to not only how to blend the flavor, but there are all kinds of measurements, what is the richness of the vaping, how much vaping comes out of the taste, is to measure all kinds of physical indicators, which are definitely with special meanings, they determine a uniqueness for each machine. They give it a very anthropomorphic title or name, what is this? The Terminator, that is Napoleon, and so on. It must be noted that these behaviors only for the core players.	Unique Play<br><br>Indicator Measurement<br><br>Nicknaming	Culture meaning	Concretization<br><br>Subcultural characteristics	Non-specialized - Specialized<br><br>Common products - labeled products<br><br>Fragmented experience - Evaluation system

the same as I do in Europe. Europe is forbidding members within EU countries to sell poppers, which they believe have a similar function because it has a more varied flavor and an inconspicuous tar feel, and as a result it attracts young people to overuse it, and other countries can observe such practices. But for e-cigarettes, I have not seen, at least Sweden has not seen the adoption of a similar policy. So, China's policy is still a little early, although it is a little imitation of some of the previous strategy, but new policy is quite obvious, they do not want to attract young people to vaping.	Compare<br><br>Points in common between poppers and e-cigarettes<br><br>Youth overuse	Policy Perspective	Restrictive policies<br><br>Purpose: To reduce the use of young people	No—Have
In 2017, others sent me such a thing (vaping device), my high school classmate sent me, he also vaping, and he would say to me using this to quit smoking, but in fact, we all know is joking, he just share a good thing with me. At that time, I remember at that time electronic cigarette is not a particularly popular thing, in the television had some sold electronic cigarette advertising.	Smoking Cessation<br><br>Entertaining Publicity and promotion<br><br>Reasonable selling	Social Dimension	Source Value Rational	Alternative - Entertainment Don't know - Know

Then, we followed the coding logic process and performed secondary coding based on the primary coding. For example, the interviewee Hetian's view that "(e-cigarette) substitution is not necessarily, tobaccos have an important and already recognized role that distinguishes them from e-cigarettes" was further analyzed for its concretized connotations, supporting logic, action strategies, and results.

- Concretized connotation (context)

"On September 19, the school staff chorus competition began, and I led the school of international relationships. Before the performance, we were waiting at the bottom of the building, the male teachers went out to smoke, and then I also went out to smoke there; originally, I did not have much communication with the teachers. The male teachers may think that I am a kid as a coach, always feel that there is no way to communicate very smoothly, and When I took out tobacco, we immediately chatted together. Cigarettes are an excellent tool for communicating with people who are older than me. I think they will confirm that you are their person at that moment, but I can still imagine that if I take out the e-cigarette, people will probably think that it is still a child using it."

- Support logic

A sign of maturity and growing up as a child is the ability to accept some level of pain (tar sensation), reflected in being able to tolerate and bear choking on something. The use of e-cigarettes, on the other hand, does not create an emotion of recognition in older people.

- Action Strategy

Hetian would use traditional tobaccos to communicate with middle-aged men between choral programs. He mentioned.

"It generally feels like smoking is a time when the relationship between two people may not necessarily be honest, and the conversation includes flattery and lies, but it always feels like the situation makes people a little more intimate. Intimacy does not necessarily mean frankness. We feel that the relationship is drawn closer."

- Results

E-cigarettes are not socially available across all age groups, and traditional cigarette use is considered a symbol of maturity among middle and young adults.

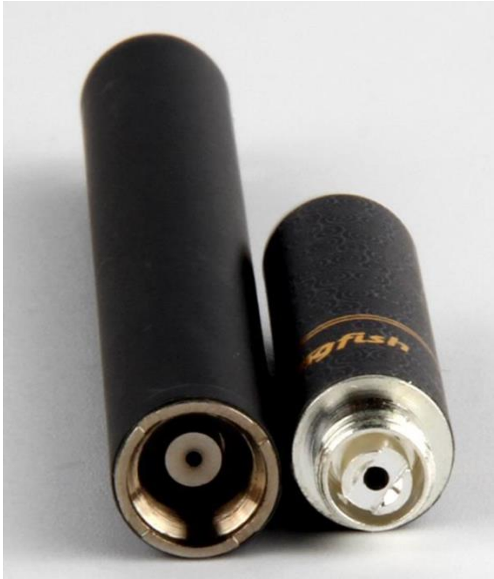

Figure S1 cigarettes device, which is the smoking cessation alternative,

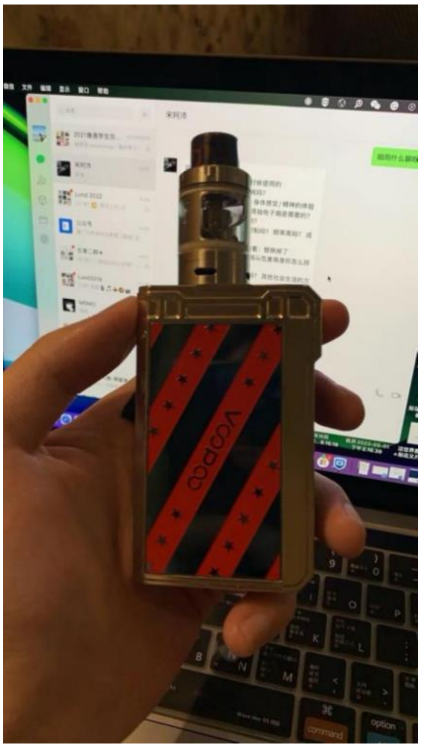

Figure S2 older e-cigarette device which needs to be lighted

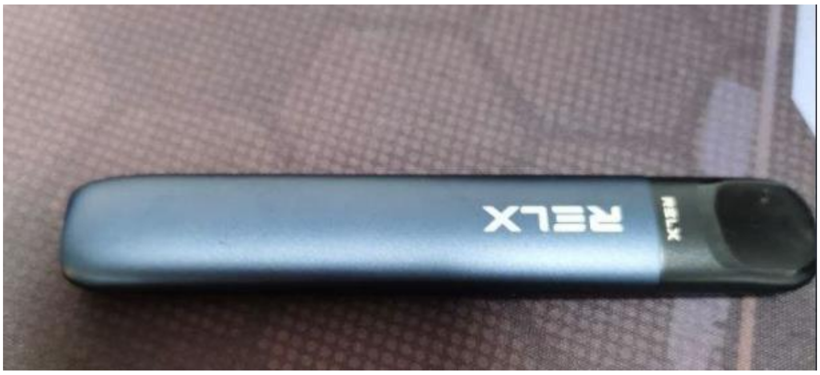

Figure S3 The latest version of lightweight, battery-operated electronic cigarettes
